# Addressing oral health equity through community service-learning and person-centered care in Ontario: patient and provider perspectives

**DOI:** 10.1371/journal.pone.0334089

**Published:** 2025-10-10

**Authors:** Abbas Jessani, Giuliana Gadoni Giovanni Borges, Jacqueline Torti, Zachary Hollingham, Trixie Patricia Vos, Natasha Roden, Sarah McLean

**Affiliations:** 1 Department of Dentistry, Schulich School of Medicine and Dentistry, Western University, London, Ontario, Canada; 2 Department of Epidemiology and Biostatistics, Schulich School of Medicine and Dentistry, Western University, London, Ontario, Canada; 3 Department of Medicine, Schulich School of Medicine Dentistry, Western University, London, Ontario, Canada; 4 Centre for Education Research & Innovation, Schulich School of Medicine & Dentistry, Western University, London, Ontario, Canada; 5 Oxford County Community Health Centre, Woodstock, Ontario, Canada; 6 Department of Anatomy and Cell Biology, Western University, London, Ontario, Canada; Shahid Beheshti University of Medical Sciences School of Dentistry, IRAN, ISLAMIC REPUBLIC OF

## Abstract

This study aimed to investigate the influence of the Community Service-Learning (CSL) program at Schulich Dentistry on the experiences and perceptions of patients and healthcare providers (HCP) at the Oxford County Community Health Centre (OCCHC) in Ontario, Canada. The CSL program aimed to address the oral health needs of equity-deserving populations and provide dental learners with experiential, community-based training. A qualitative research methodology using a Community-Engaged Research (CEnR) framework was employed. Data was collected through one-on-one interviews with 21 patients and six HCPs at the OCCHC. Inductive thematic analysis was conducted to identify key themes. As a result, five main themes were identified, with overlap between patients and HCPs. Two major themes emerged from the interviews with patients (1) challenges and barriers to dental care and (2) enhanced access to dental care through the CSL program; highlighting stigma and discrimination due to public dental insurances and low socioeconomic status. From the HCPs’ perspectives, (3) person-centred care was the main identified theme, emphasizing the importance of understanding patients’ individualized circumstances and social determinants of health when providing dental care. Additionally, common themes between patients and HCPs were also identified as (4) supportive environment and (5) patient empowerment and self-confidence. In conclusion, the CSL program addressed the oral health needs of equity-deserving patients by improving patient access to dental care while increasing patients’ self-esteem and confidence through a person-centred care approach. These findings highlight the importance of community-integrated models of dental care in addressing oral health inequities and training future dental professionals.

## Introduction

Community Service-Learning (CSL) in dentistry is an experiential learning approach that integrates meaningful community engagement with academic learning and professional skill development [1]. In dental education, CSL plays a critical role in enhancing learners’ understanding of barriers and the socio-cultural determinants of oral health that influence patient outcomes [2]. Consequently, engaging the community for didactic and experiential training is essential for understanding and addressing the barriers to accessing oral health [3].

The literature reveals a variety of terms used to describe community education programs, reflecting the diverse models, goals, and contexts in which these programs are implemented [4]. Despite the variation in terminology ranging from service-learning to community-based education and outreach these approaches share a common goal of integrating community engagement into the educational experience.

In this study, Community Service-Learning (CSL) is implemented using key principles drawn from the Yoder Framework [5]. Through its evidence-based and systematic approach, this model is designed to immerse dental learners in real-world community settings, enabling them to provide dental care while gaining firsthand insights into the social determinants of health and systemic barriers faced by socially marginalized populations.

By engaging learners in longitudinal care, CSL fosters continuity in patient-provider relationships and encourages the development of critical skills such as cultural humility, empathy, and interprofessional collaboration, as stated in Jessani et al., 2025 [5]. The approach not only enhances learners’ clinical training but also promotes a holistic and socially responsive understanding of oral health. Through CSL, learners are given the opportunity to engage meaningfully with the community, reflect on their experiences, and integrate academic knowledge with practical, community-informed learning [5]. This reinforces the importance of preparing future dental professionals who are not only clinically competent but also socially conscious and committed to advancing health equity [5]. Furthermore, CSL pedagogy nurtures a sense of empathy and cultural humility, preparing learners to address the oral health needs of diverse equity-deserving patient populations [3,6]. Equity-deserving populations are socially marginalized individuals facing psychosocial disparities and barriers while accessing oral health services [7]. Some of the common groups include low-income families, rural community residents, ethnic minorities, individuals living with HIV, high-risk youth experiencing housing instability and addiction, refugees and new immigrants, Indigenous peoples, and Two-Spirit, lesbian, gay, bisexual, transgender, queer (or questioning), and other sexual and gender minorities (2SLGBTQ+) people and others [7]. The CSL pedagogy enhances patients’ ability to navigate challenging systems while equipping learners with the knowledge to address the ethical and logistical complexities of professional practice [8].

Beyond its educational intent, CSL is instrumental in promoting equity in dental care. By prioritizing service to equity-deserving populations, CSL programs address disparities in oral health access and outcomes [9]. This approach not only improves oral health in these communities but also builds trust and bridges systemic gaps, fostering a more inclusive and compassionate dental care system [10].

### CSL at schulich dentistry

The CSL program at Schulich Dentistry, Western University, was implemented in the academic year of 2021−2022 in the third (D3) and fourth (D4) year undergraduate dental program [3]. The CSL program aims to: 1) address the high unmet oral health treatment needs of equity-deserving community members; and 2) provide undergraduate dental learners with experiential, community-based training emphasizing person-centred and trauma-informed care [3]. In D3, learners rotate through various community organizations that provide social and healthcare services to underserved populations in Southwestern Ontario. Clinical screenings are performed at these community organizations, and patients are offered one course of free treatment at the Schulich Dental Clinic. In D4, mandatory rotations are assigned to dental clinics at the Oxford County Community Health Centre (OCCHC) to address the unmet oral health needs of equity-deserving community members. The development and integration plan for the CSL program has been presented elsewhere [3]. During the 2021−22 academic year, D4 learners were placed at the OCCHC in Woodstock, Ontario, to address the high oral health treatment needs of the equity-deserving populations within the Oxford County region [3]. Although the CSL program was implemented for D3 and D4 learners, this paper will specifically focus on the impact and experiences of the patients accessing the CSL program at the OCCHC. The OCCHC is a multi-disciplinary health centre that provides primary care, health promotion, and community development programs, particularly for low-income and socially marginalized individuals [3]. The priority populations they serve are persons with addictions, persons with mental health issues, low-income families, at-risk youth, and isolated seniors. Regarding those seeking dental care, the OCCHC prioritize community members without dental benefits and those who do not qualify for government dental programs [3].

The CSL program for D4 undergraduate dental learners operates in collaboration with OCCHC’s dental professionals, who also provide oral healthcare services through a seniors’ dental program. The CSL program complements these services by offering comprehensive dental care on Wednesdays through Fridays, while the seniors’ dental program operates on Mondays and Tuesdays. Participating dental learners work in groups of four and complete four-week rotations at the OCCHC, where they are supervised by community dentists. Evaluation is conducted through both summative and formative assessment methods. Learners are assessed on their clinical competencies across various aspects of person-centered dental care. In addition to clinical skills, they are evaluated on behavioral and professional attributes, including communication, interpersonal conduct, empathy and perspective-taking. Henceforth, learners are required to submit reflective journals that incorporate structured thematic reflections and experience-based narratives. These reflections are intended to encourage critical thinking and self-assessment based on their clinical experiences at OCCHC.

The goal of this research was to investigate the experiences and perceptions of patients and healthcare providers (HCPs) engaging with the CSL program at Schulich Dentistry. While the learners experience remains a crucial aspect of CSL, this study deliberately centers on patients and HCPs to shift focus from a learner-centered approach which often prioritizes educational outcomes to one that foregrounds the needs, insights, and lived experiences of those receiving and facilitating care [11]. By doing so, this research positions the community as a teacher, emphasizing the reciprocal nature of learning and the importance of understanding CSL’s broader impact beyond learners perspective.

## Methods

Ethics approval was granted by the Western University Health Sciences Research Ethics Board (Study Number: 2023-122186-79114). The study employed a Community-Engaged Research (CEnR) framework, an approach that emphasizes meaningful collaboration between researchers and community members throughout the research process [12]. This methodology goes beyond traditional research paradigms by actively involving the community in shaping the research agenda, co-developing study design, collecting and interpreting data, and disseminating findings. CEnR is rooted in principles of mutual respect, co-learning, and shared decision-making, recognizing the unique knowledge and lived experiences that community members bring to the table.

In this study, the CEnR approach included partnering specifically with underserved and historically marginalized patients who are often excluded from academic research due to structural barriers or systemic neglect. By engaging patient partners and community stakeholders as equal partners rather than passive subjects, the research aimed to promote equity, increase the relevance and cultural sensitivity of the findings, and ultimately ensure that the outcomes benefit those most affected. This inclusive and participatory model not only enhances the quality and impact of the research but also fosters trust, empowerment, and capacity-building within the communities involved [13].

### Participant recruitment and data collection

Following the completion of treatment by the dental learners, patients were invited to participate in one-on-one interviews. The interviews aimed to explore patients’ experiences with unmet oral healthcare needs, barriers to accessing oral healthcare services, and their experiences in the CSL program at the OCCHC. Purposeful sampling was used to recruit participants who have not only received care from the CSL program but were available and willing to participate [14]. This was conducted through various methods, including online advertisement on the OCCHC’s social media platforms and by directly contacting patients who have either currently or previously received care from the CSL program at the OCCHC. A demographic survey was administered to patient-participants prior to their one-on-one interview to understand the context of the participants’ perceived experiences.

HCPs who work at the OCCHC were also recruited to participate in one-on-one interviews. These interviews aimed to explore HCPs’ perspectives on the unmet oral health needs and barriers to accessing oral healthcare services among service users at the OCCHC, as well as the care received by the CSL program at the OCCHC on patients. Purposeful sampling was also used to recruit HCP participants. This was conducted by directly contacting those who provided services to patients who have either currently or previously engaged in the CSL program at the OCCHC.

Each interview was audio-recorded and transcribed, and all transcription of raw data was completed by a team member soon after the data collection. By following the CEnR approach, OCCHC stakeholders (ZH and TV) and a peer-facilitator (NR) were actively engaged and participated in participant recruitment, data collection and, subsequently, data analysis and knowledge creation. The stakeholders and peer-facilitator were recruited based on their in-depth understanding of the participant population.

### Analysis

**Fig 1:**
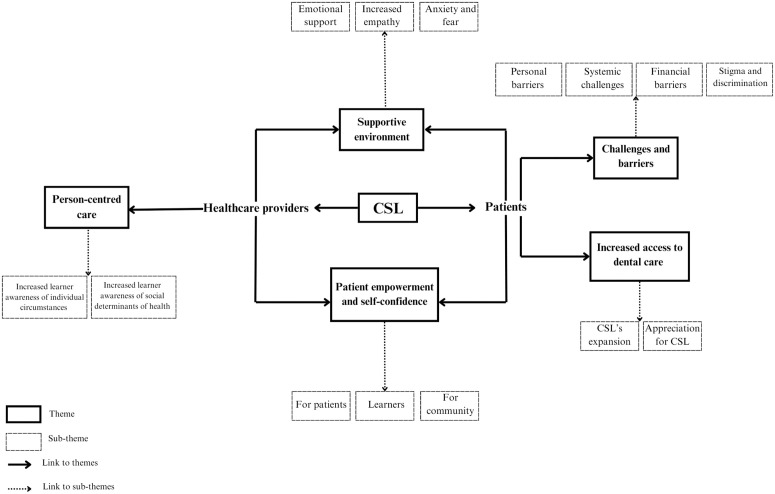
Interactive mapping of the themes and sub-themes from patients and HCPs.

As adapted from Schwab et al., [15] the study adhered to a repeated six-step process to carry out inductive thematic analysis. The initial step entailed researchers (AJ and GB) independently reading every transcript cautiously to gain familiarization with the content while coding the initial fundamental thoughts. The second step involved a peer-review of the codes, followed by the researchers mutually concluding on a finalized coding of all the transcripts. The third step required one member (GB) to consolidate similar codes to identify the significant and repetitively mentioned ideas that relate to the research objectives. In the fourth step, team members (AJ and SM) amended and recombined comparable codes to generate the main themes. The fifth step consisted of the refining and naming of the dominant themes. Lastly, the sixth step produced a theme map, visually connecting the codes and main themes as done by Brondani [8] (Fig 1). In line with the CEnR approach, stakeholders from the OCCHC (ZH and TV) and a peer-facilitator (TR) were engaged in member checking and confirming the accuracy of the themes.

### Trustworthiness

A variety of strategies were used to ensure that trustworthiness which evaluates the overall quality, credibility, and confirmability of qualitative research was upheld through the study [16]. Our interdisciplinary research team includes scholars and trainees with expertise in dental, medical and social sciences, as well as community-engaged learning. This composition, coupled with the active involvement of our community partners from OCCHC, including the executive director, a former service user (peer-facilitator), and a staff member, helps to ensure our research is both academically rigorous and grounded in the realities of the community it serves.

The CEnR framework encouraged ongoing collaboration between the research team, stakeholders from the OCCHC, and the peer-faciliator—a crucial step in shaping a protocol that reflects the needs of the community. Additionally, a peer-facilitator, with lived experience of accessing services from the OCCHC and care from the CSL program, provided regular feedback on aspects of the study, including the study objectives, interview guide, interview discussions, and results and conclusions. After completing the coding and thematic analysis process, credibility was ensured by holding debriefing sessions through the interview process as a means to review the participants’ perspectives. A debriefing session was also held following the initial analysis to ensure that the results accurately reflect the perspectives of the participants. Confirmability was achieved by conducting multiple coding processes and debriefing sessions with OCCHC stakeholders and the peer-facilitator to ensure that the results fully reflected participants’ experiences [17]. Lastly, data saturation was achieved when no new themes or patterns emerged, ensuring that the data was thoroughly analyzed and reinforcing the strength and trustworthiness of the findings [15].

## Results

A total of 21 participants completed a demographic survey; *n* = 21 patients, and a total of 27 participants completed a one-on-one interview; *n* = 21 patients and *n* = 6 HCPs. Eleven male and 10 female patients, with an average age of 48 years, participated in the patient interviews (Table 1). Seven patients reported having post-high school education and eight reported having full-time or part-time employment (Table 1). Of the six HCPs, two were directors of the primary care services, while others included a community outreach worker, a family physician, a dental assistant, and a nurse practitioner. They had all been employed at the OCCHC for a minimum of two and a maximum of thirteen years.

**Table 1:** Demographic characteristics of patients (N = 21).

Variable	Frequency, n (%)
Gender	
Male	11 (52.4%)
Female	10 (47.6%)
Age (years), mean	47.9
Ethnicity	
White	21 (100%)
Other	0 (0%)
Highest educational level	
Less than high school or high school diploma or equivalent	14 (66.6%)
Higher education (post-high school)	7 (33.4%)
Employment status	
Employed (part-time/ full-time)	8 (38%)
Un-employed/ welfare and disability insurance	8 (38%)
Other (retired)	5 (23.8%)

Table 2 summarizes the results from a pre-interview survey, which was administered to patients to gather data on characteristics relevant to dental care and the context of their participation in the CSL program. The questionnaire was based on questions from the validated Canadian Health Measures Survey (CHMS) and is available in S1 Appendix.

**Table 2:** Self-perceived oral health and access to dental care (N = 21).

Variable	Frequency, n (%)
Self-perceived oral health condition	
Excellent/Very good/ Good	11 (52.4%)
Fair/Poor	10 (47.6%)
Importance of oral health care	
Very important/important	21 (100%)
Somewhat important/not at all important	0 (0%)
Regular medical doctor	
Yes	20 (95.2%)
No	1 (4.8)
Regular dentist	
Yes	3 (14.3%)
No	18 (85.7%)
How would you feel if you had a dentist appointment tomorrow?	
Extremely to fairly anxious	16 (76.1%)
Not anxious	5 (23.8%)
Dental visit (prior to CSL Progarm)	
Less than 1 year to 4 years ago	8 (38.1%)
4 years ago or more	13 (61.9%)
Stigma and discrimination by dental professionals	
Yes	6 (28.6%)
No	15 (71.4%)
Avoiding dental care due to cost (prior to CSL program)	
Yes	16 (76.2%)
No	5 (23.8%)

The major themes derived from the patient interviews were (1.1) challenges and barriers, (1.2) enhanced access to dental care. The major theme from the HCP interviews was (2.1) person-centred care. The results revealed the common themes between patients and HCP interviews, such as (3.1) supportive environment and (3.2) patient empowerment and self-confidence. Fig 1 provides the thematic map of the themes that constitute our study findings.

The majority (*n* = 18) of patient participants reported not having regular access to a dentist. Thirteen patients had not had a dentist appointment for more than four years prior to getting accepted to the CSL program. Having dental anxiety and avoiding dental treatment due to cost was reported by sixteen patients, while fifteen patients reported experiencing stigma and discrimination in their prior experience with dental professionals.

### 1. Patient interviews

Table 3 (S2 Appendix) provides a summary of the themes, sub-themes, and codes, with illustrative examples from patient interviews.

#### 1.1 Challenges and barriers to dental care.

Challenges and barriers pertinent to accessing dental care were often revealed by patients. Four subthemes were identified and related to A) financial, economic, and employment-related constraints, B) systemic challenges, C) personal barriers to care, and D) stigma and discrimination. Finances and economics were among the important barriers to accessing dental care. Patients reported ‘*hardships’* and ‘*financial constraints’* when referring to paying for dental treatment ‘*out of the pocket.’* As one patient mentioned:

*“Financial is always one of the first things (barriers). I’m on ODSP (Ontario Disability Support Program -* is a social assistance program to help people with disabilities who are in financial need. ODSP typically covers basic dental care, including cleanings, fillings, extractions, X-rays, and emergency treatments)*, and it wasn’t until fairly recently that ODSP started covering more.”* (P5)

This highlights the direct impact of limited financial resources on patients’ ability to afford dental care. The reliance on programs like ODSP and the fact that coverage has only recently improved underscores the systemic challenges faced by vulnerable populations in obtaining adequate oral healthcare.

The patients also deemed a consistent employment opportunity with dental insurance very important to maintain their oral health. One patient’s experience illustrates this connection:

*“When I started working and didn’t have dental care, that’s when I started having problems...my teeth started going even worse because I had to let them go because I couldn’t afford the bills. My teeth started having cavities, and then they started falling apart because I couldn’t go and get them redone.”* (P6)

This narrative reveals a link between employment status, insurance coverage, and oral health outcomes. The lack of insurance can lead to a rapid decline in oral health, creating conditions that are difficult to reverse when care is finally provided.

Beyond financial barriers, patients also described experiencing stigma and discrimination within dental care settings. One participant mentioned being turned away by multiple dental offices due to their reliance on social assistance:

*“They (dental offices) just shun me out basically they just turn you away...I’ve gone to probably about a handful of dentists. And they all turned me away due to my OW (Ontario Works).”* (P17)

Another patient confirmed this, stating

*“They (dental offices) don’t like seeing patients on government plans.” (P19)*

These experiences highlight a systemic bias against individuals relying on government assistance, suggesting that access to dental care is not only a matter of affordability but also of social stigma and discriminatory practices within the healthcare system. The effect of these barriers creates an environment where patients feel unwelcome and are denied care based on their socioeconomic status and source of income. Overall, patients expressed their dissatisfaction with accessing private dental offices with government insurance plans.

#### 1.2. Increased access to dental care through the CSL program.

Increased access to dental care through the CSL program was noted by many patients. Two subthemes were identified and related to: A) appreciation for CSL and B) the need for CSL’s expansion (Table 3 in S2 Appendix). Patients reported visiting the dentist for the ‘*first time in several years’* and noted being *‘grateful’* for the ability *‘to smile’* due to the dental treatment they received through the CSL program.

Beyond the functional improvements in their oral health, patients reported feeling ‘*safe*’ and ‘*cared fo*r’ by the dental learners, who were characterized as ‘*compassionate*’ and attentive to their needs. As one patient stated:

*“It’s a very welcoming place...Everybody’s compassionate towards you. So it’s a good place to be...They took good care of me.”* (P2)

This statement underscores the importance of a positive and supportive clinical environment in fostering patient trust and engagement, particularly for individuals who may have had negative experiences in the past.

The positive impact of the CSL program extended beyond the immediate dental treatment, with patients expressing a deep appreciation for the holistic care they received. One patient shared this satisfaction:

*“I love this place (OCCHC/CSL program). I recommend this to everybody even to my enemies...everything’s good. You know I have everybody in here works so hard...I’m just quite satisfied with the way it is.”* (P12)

This shared perception among patients speaks to the comprehensive nature of the care provided, and the dedication of the CSL team. Patients also suggested that having access to the clinic had ‘changed their life’ and/or significantly supported their overall ‘well being.’ As one patient explained:

*“The students working in community clinic, it’s not helping just the dental side, it’s helping us all around because it’s not just about your teeth, it’s really not. It’s your mental health, there’s your physical health, there’s counseling, there’s other support that you need.”* (P5)

This finding highlights that oral health is intimately connected to overall health and well being, and that the CSL program, by addressing dental needs, is also contributing to improvements in patients’ mental and physical health, and their access to other essential support services. Considering their positive experience, they further expressed their desire to expand the CSL program:

*“The biggest thing I would suggest is to expand it. It would need to expand, however that looks. I would be willing to speak for fundraising.”* (P2)

This desire for expansion highlights the perceived value of the program within the community, and the recognition that many more individuals could benefit from its services.

### 2. Healthcare providers

Table 4 (S3 Appendix) summarizes the themes, sub-themes and codes identified from the interviews with HCPs, including verbatim examples.

#### 2.1. Person-centred care.

The HCPs highlighted the aspect of person-centred care in the CSL program. Two subthemes were identified: A) increased learner awareness of individualized circumstances and B) increased learner awareness of social determinants of health (Table 4 in S3 Appendix).

During their interviews, HCPs underscored the importance of dental learners developing a connection with the patients while acknowledging individual factors that impact their oral health outcomes. As one HCP noted:

*“I’m hopeful that the dental students get this perspective and understand the complexities of substance use concerns and mental health and how that plays a role in their (patient’s) dentition.’’* (HCP3)

This reflects a desire for learners to move beyond a clinical approach and appreciate the ways in which patients’ social and behavioral contexts can impact their oral health. Another HCP stated:

*“I think it broadens their (learners’) horizon, their awareness of the kind of challenges that folks encounter’’* (HCP6)

Here, the HCP suggests that the CSL program plays a crucial role in expanding students’ understanding of the diverse challenges faced by vulnerable populations, fostering a greater sense of empathy in their practice.

HCPs suggested that the CSL program was an ‘*eye-opening’* experience for learners. It was perceived as a ‘*unique education opportunity*,’ ‘*shaping their dental and social knowledge’* by expanding their interactions with patients from various backgrounds. One HCP explained:

*‘‘I think it has opened their (learners’) eyes to the severity of dental needs. And I’m hoping they do take this time to experience the challenges and barriers that people do have and that when they get into practice they have to understand that not everybody is the same.’’* (HCP4)

This underscores the potential of CSL in challenging students’ preconceived notions and developing an understanding of the social factors that influence oral health disparities. By directly engaging with patients facing complex challenges, learners gain invaluable insights that can inform their future clinical practice and promote more equitable care.

This experiential learning enables learners to work with patients from equity-deserving backgrounds, providing perspective-taking and increased awareness towards social determinants of health. As one HCP mentioned:

*‘‘…it’s systemic and there’s a lot of factors that are at play. Many of my clients experience trauma throughout their childhood or young adulthood that they have major mental concerns and difficulty accessing care for that.”* (HCP3)

This observation highlights the connection of oral health with broader social and systemic issues, such as trauma and mental health, and emphasizes the need for a holistic approach to care that addresses these underlying factors. HCPs highlighted the importance of a comprehensive approach to dental care, acknowledging the effect of social and systemic factors that should be considered when treating patients, as stated:

*‘‘What I really hope is that it changes our perspective or our lens of practise. And sometimes, society kind of puts this blame on clients to use drugs and that it’s their choice, but I really want them to understand that it’s a chronic illness, it’s a chronic disease.’’* (HCP4)

This statement illustrates the need for a change in perspective among dental professionals toward a deeper understanding of the social and economic factors that contribute to oral health conditions. The CSL program, by exposing learners to these realities, can play an important role in fostering this shift and promoting more equitable dental care.

### 3. Shared themes

Table 5. (S4 Appendix) provides a summary of the shared themes, subthemes, and codes from patients and HCPs, and excerpts from the interviews as examples.

#### 3.1. Supportive environment.

Both HCPs and patients appreciated the supportive environment for the delivery of dental care through the CSL program. Three subthemes were identified: A) increased empathy, B) emotional support, and C) dealing with anxiety and fear (Table 5 in S4 Appendix).

Many patients reflected on the supportive care they received from the dental learners, noting a significant difference from previous dental experiences. As one participant shared,

*“I feel a lot more comfortable here...they’re not just roughing and like pulling things out shoving things in here...I feel like these kids genuinely want to learn what’s going on with my mouth.”* (P11)

This statement reveals a sense of vulnerability among patients, and the importance of being treated with respect. Patients felt that the dental learners were not only focused on the technical aspects of the treatment, but also showed a genuine interest in their overall well-being. Another patient emphasized this feeling of ease and comprehensive care, stating,

*“I think the students did a great job... they made you feel at ease. They told you exactly what they were going to do, and it’s more of a care.”* (P7)

This highlights the importance of clear communication and patient-centered care in creating a positive and supportive environment, fostering a sense of trust and safety.

The HCP narrated similar aspects of developing trust and empathy toward patients. They perceived empathy as a way of facilitating treatment and learning, as shared by the healthcare provider:

*“I think by them (students) having a little more empathy, they’ve realized that by taking that extra time that patients do respond to them because they’re treated as a person.”* (HCP4)

This observation underscores the importance of empathy in building connections with patients and how this, in turn, leads to improved patient outcomes and a more positive learning environment for the learners. The HCPs suggest that when learners demonstrate empathy, patients are more likely to be receptive to treatment.

Patients also mentioned the assistance in dealing with mental health such as anxiety and how the environment of CSL helped them navigate the *fear* of dental appointments, as shared by one patient:

*“I suffer from a lot of anxiety... but when I came here, I didn’t have a blood pressure problem for the first time.”* (P8)

This exemplifies how dental care delivery is not only related to clinical procedures but also to developing trust and relationships between providers and patients. Additionally, it is evident how CSL is perceived as an optimal environment for building empathy and how it positively affects service users’ experiences.

#### 3.2. Patient empowerment and self-confidence.

Both patients and HCP revealed CSL’s positive impact through patient empowerment and self-confidence. Patients identified improved self-esteem and well-being after receiving dental treatment through the CSL program. Increased oral health awareness, community engagement, and access to comprehensive dental care were the main positive aspects of CSL’s community impact (Table 5).

The transformative effect of the CSL program on patients’ self-confidence is evident in their own words. As one patient shared,

*“It has built up my self-confidence so much like when I’m out, I’m not like [mouth closed gesture]. You know, and I’m not afraid to smile and say hi.”* (P1)

This statement illustrates how adverse oral health conditions can negatively impact social interactions and self-perception, and how the CSL program helped to address this issue, enabling patients to engage more confidently in their daily lives. Another patient reinforced this sentiment, stating,

*“It’s giving me confidence that my smile is going to be better...I do feel a lot better now...I don’t have to worry about that tooth ever getting really infected or anything.”* (P13)

This quote highlights both the psychological and physical benefits of receiving dental care. Beyond the cosmetic improvements, patients also experienced relief from the anxiety and fear associated with untreated dental problems.

HCPs also mentioned the improvements they perceived in their patients

*“The dental program has been life-changing for my clients and I am just so excited to see where it leads and the growth.’’* (HCP2)

This observation from an HCP reinforces the idea that the CSL program is not just providing dental treatment, but has a relevant role in the lives of patients.

Additionally, patients perceived the CSL program as an important, practical and hands-on learning experience for students, and patients mentioned being happy to help in their education. Both HCPs and patients stated that the program can broaden prospective professional opportunities and improve their skills in dealing with people from different backgrounds. Participants shared that:

*“It leads them into different job opportunities (…) so they can broaden their patients instead of just looking at one group.”* (P4)

*“You’re kind of like a live demonstration of what they’re learning...it’s actually like hands-on right there...they’re physically able to do the work and learn as they go just like that.”* (P13)

*“This kind of fills all those gaps for them and gives them a tangible way a career path maybe through public health or through volunteerism.”* (HCP6)

These findings indicate that patients see the CSL program as beneficial not only for themselves, but also for the learners, as it exposes them to a wider range of patients and prepares them for diverse professional opportunities. They also highlight the unique opportunity the CSL program provides for learners to gain clinical skills in a real-world setting, while simultaneously providing much-needed care to the community.

The program was also perceived as a ‘*reliable’* and ‘*very important’* source of healthcare, increasing the community’s access. They appreciated that the program is part of a primary health and community setting:

*“It’s much needed in our community. Being at the community health centre it’s like the perfect location. Because again, our vulnerable clients do visit that location. It’s accessible. It’s easy to get to.”* (HCP3)

This underscores the importance of the CSL program’s location within the OCCHC, which makes it easily accessible to vulnerable populations who may face significant barriers to accessing traditional dental care settings.

## Discussion

Our study aimed to investigate the role of the newly implemented CSL program on the experiences and perceptions of patients and HCPs. All the patients who accessed the CSL program identified as cisgender and most of them had not seen a dental professional in more than four years prior to their engagement with the program. Most of the patients accessing the OCCHC and CSL programs did not have regular access to dental care, highlighting the vital role of the CSL program in addressing real health disparities and promoting equity for underserved community members. Patients also reported experiencing stigma or discrimination by their previous dental care providers and reported cost as the primary barrier to seeking dental treatment. These findings highlight the severe lack of access to basic dental care in Southwestern Ontario, particularly in the Oxford County region, which includes a significant equity-deserving population; hence, there is a need to enhance access to dental care services in this region [18].

Several significant barriers to accessing dental care emerged from our analysis, including financial constraints, systemic challenges, and stigma and discrimination. Participants highlighted financial barriers such as the high cost of dental treatment and the lack of dental insurance as key obstacles to accessing dental care services. Additionally, public dental insurances such as OW and ODSP were also reported as key barriers to accessing dental care. These financial and resource-related barriers are critical social determinants of health that disproportionately affect equity-deserving populations, limiting their access to essential dental services [19,20]. Addressing these barriers through the expansion of services via the CSL program, as highlighted in our findings, can help mitigate these inequities.

Stigma and discrimination by dental care providers based on patients’ social status also emerged as an important theme. These factors can erode trust, further limiting access to dental care and contributing to high levels of unmet treatment needs, as reported by Schwab et al. [15]. Public dental insurances such as OW and ODSP further perpetuated this stigma as participants noted that dental professionals showed reluctance in providing care to patients with these coverages. The CSL program, with its robust didactic and experiential components, has the potential to train future dental care providers to recognize and address stigma and discrimination [21]. CSL, where learners learn from the lived experiences of equity-deserving community members and provide person-centred dental care in safe and supportive spaces, can play a pivotal role in fostering cultural safety and reducing stigma in dental practice. Person-centred care emerged as a key theme identified by healthcare providers. Through the CSL program, patients received care tailored to their individualized needs and circumstances, fostering positive dental care experiences, as reported by Taylor and colleagues [22].

Common themes emerged from both patients and healthcare professionals regarding the CSL program. The program fostered a supportive environment for all participants, with patients reporting empathetic and emotionally supportive interactions with learners and staff. These interactions contributed to a sense of ease and a reduction in dental anxiety among patients. Evidence suggests that positive and empathetic behaviour exhibited by dental care providers enhances patient access to care and facilitates the development of trust-based relationships [23].

Access to dental care through the CSL program significantly improved self-confidence, enabling community members to ‘*smile again*.’ It empowered patients to take ownership of their oral health, fostering improved self-esteem. Behar-Horenstein and colleagues similarly reported that CSL programs can empower equity-deserving communities to take charge of their oral health [24]. Through the delivery of person-centered care and consistent oral health education, service learning has the potential to generate positive patient outcomes and experiences, ultimately contributing to improved oral health and overall well-being at both individual and community levels.

Our results have several important implications. They highlight the need for community-based dental care tailored to the equity-deserving populations who often face high unmet dental treatment needs due to personal barriers (e.g., financial constraints, lack of education) and structural barriers (e.g., stigma and discrimination). In diverse countries like the United States and Canada, with high rates of immigration from multicultural and multiethnic backgrounds, it is essential to train dental care professionals within the framework of community-integrated models [25]. These models address the unique oral health needs of the communities and populations they serve.

Specifically in Canada, with the introduction of the Canadian Dental Care Plan, a semi-public healthcare system promising essential dental care for underserved individuals -particularly those from low socioeconomic status, new immigrants, gender and sexual minorities, individuals with disabilities, and seniors, it is more crucial than ever to provide experiential learning opportunities for dental learners [26,27]. Such training prepares them to address the unmet dental treatment needs of future patient populations effectively. This approach not only ensures equitable oral health care for equity-deserving populations but also offers comprehensive training for future dental care providers, fostering empathy, cultural humility, and community-centred care [3].

Our study has limitations. First, the relatively small sample size of patients and HCPs may impact the transferability of our findings. While qualitative research provides rich, in-depth insights, future studies should incorporate larger quantitative metrics to provide a more comprehensive understanding of the unmet oral health needs and experiences with the CSL program. Further, some participants may have been uncomfortable sharing their stories, despite our best efforts to make the environment as safe as possible.

Moreover, while the CSL program at Schulich Dentistry attempts to address disparities in oral health access and outcomes, it is important to acknowledge its current context. The program, as described, provides free-of-cost treatment to all patients accessing OCCHC, providing them with a safe dental home to address their high unmet oral health dental treatment needs. As a newly implemented initiative, the program’s long-term impact, including its potential for systemic change, is still emerging. Therefore, while the CSL program shows potential to reduce barriers to care for equity-deserving populations, claims regarding its impact on significant disparities should be interpreted cautiously, with an understanding of the program’s initial stages of implementation and evaluation. Future research could also explore the students’ experience to further contextualize the program’s impact. Another limitation of our study is the demographic homogeneity of the patient sample with all the study participants being Caucasian. This restricts the transferability of our findings to more diverse populations such as Black, Indigenous and People of Color who may experience different healthcare challenges and outcomes.

Finally, while programs such as CSL at Schulich Dentistry offer valuable experiential learning and address the oral health needs of equity-deserving community members, it is important to acknowledge potential critiques of service-learning approaches in dental education. Rivkin-Fish, 2011 [28] argues that these programs may foster a ‘paternalistic ethic’ among dental learners, where learners may not fully address and acknowledge the structural inequalities that create barriers to care. Additionally, concerns have been raised about whether dental student volunteerism might be more apparent than advocating for systemic changes, such as universal coverage and prioritization of preventive actions [29]. Therefore, it is crucial to consider how the CSL program can be designed and implemented to not only provide immediate care but also contribute to long-term structural changes that ensure equitable access to dental care for historically excluded populations. Future research should explore how such programs can be integrated with policy efforts to achieve improvements in oral health equity.

## Conclusions

This study highlighted the barriers to dental care experienced by equity-deserving community members in Southwestern Ontario while also emphasizing the positive role of the CSL program in addressing oral health disparities in the region. This study is the first of its kind in Ontario, Canada, to implement a new CSL program and evaluate its impact on equitable dental care, providing valuable insights for future research and program development.

## Supporting information

S1 Appendix (Survey), Demographic survey (Oxford). (DOCX)

S2 Appendix Themes, sub-themes, and codes, with verbatim examples from the patients. (DOCX)

S3 Appendix Themes, sub-themes and codes, with verbatim examples from the HCPs (N = 6). (DOCX)

S4 Appendix Shared themes, subthemes, and codes with verbatim examples from the patients and HCPs. (DOCX)
