# Supplementary material for: Addressing oral health equity through community service-learning and person-centered care in Ontario: patient and provider perspectives
Source: PLoS One. 2025 Oct 10;20(10):e0334089. doi: 10.1371/journal.pone.0334089 (PMC12513611; doi:10.1371/journal.pone.0334089)
Supplement: S1 Appendix — (DOCX) [file pone.0334089.s001.docx]

**Appendix 1** (Survey)

Demographic survey (Oxford)

**Q1** What is your gender?

**Q2** How old were you on your last birthday?

**Q3** From the following list, which racial or cultural groups do you belong to? (You may choose more than one option)

▢ White (1)

▢ South Asian (e.g., East Indian, Pakistani, Sri Lankan) (2)

▢ Chinese (3)

▢ Black (4)

▢ Filipino (5)

▢ Latin American (6)

▢ Arab (7)

▢ Southeast Asian (e.g., Vietnamese, Cambodian, Malaysian, Laotian) West Asian (e.g., Iranian, Afghan) (8)

▢ Korean (9)

▢ Japanese (10)

**Q4** What is the highest certificate, diploma or degree that you have completed?

O Less than high school diploma or its equivalent (1)

O High school diploma or a high school equivalency certificate (2)

O Trade certificate or diploma (3)

O College, CEGEP or other non-university certificate or diploma (other than trades certificates or diplomas) (4)

O University certificate or diploma below the bachelor's level (5)

O Bachelor's degree (e.g.B.A.,B.Sc.,LL.B.) (6)

O University certificate, diploma or degree above the bachelor's level (7)

**Q5** What is your current employment status?

o Employed (part-time) (1)

o Employed (full-time) (2)

o Un-employed (3)

o Welfare insurance (4)

o Disability insurance (5)

o Student (6)

**Q6** In general, how would you rate the health of your mouth? (Please choose one)

O Excellent (1)

O Very good (2)

O Good (3)

O Fair (4)

**Q7** How important do you think the health of your mouth is? (Please choose one)

O Very important (1)

O Somewhat important (2)

O Not important (3)

O I don't know (4)

**Q8** Do you have a regular medical doctor?

O Yes (1)

O No (2)

**Q9** Do you have a regular dentist?

O Yes (1)

O No (2)

**Q10** If you went to a dentist for treatment tomorrow, how would you feel?

O Not anxious (1)

O Slightly anxious (2)

O Fairly anxious (3)

O Very anxious (4)

O Extremely anxious (5)

**Q11** When was the last time you saw a dental professional (dentist or dental hygienist) (Prior to accessing dental services at the OCCHC)? (Please choose one)

O Less than 1 year ago (1)

O 1 year to less than 2 years ago (2)

O 2 years to less than 3 years ago (3)

O 3 years to less than 4 years ago (4)

O 4 years to less than 5 years ago (5)

O 5 or more years ago (6)

O Never (7)

**Q12** When was the last time you saw a dental professional (dentist or dental hygienist)? (Please choose one)

O Less than 1 year ago (1)

O 1 year to less than 2 years ago (2)

O 2 years to less than 3 years ago (3)

O 3 years to less than 4 years ago (4)

O 4 years to less than 5 years ago (5)

O 5 or more years ago (6)

O Never (7)

**Q13** Have you ever felt discriminated against by a dental professional while accessing dental care services?

O Yes (1)

O No (2)

**Q14** Prior to acceding dental services at OCCHC, have you avoided having some or all the dental treatment that was recommended because of the cost?

O Yes, because I could not afford the cost of the treatment. (1)

O No (2)
