# Supplementary material for: Addressing oral health equity through community service-learning and person-centered care in Ontario: patient and provider perspectives
Source: PLoS One. 2025 Oct 10;20(10):e0334089. doi: 10.1371/journal.pone.0334089 (PMC12513611; doi:10.1371/journal.pone.0334089)
Supplement: S2 Appendix — (DOCX) [file pone.0334089.s002.docx]

**Appendix 2**

**Table 3: Themes, sub-themes, and codes, with verbatim examples from the patients.**

| Themes | Sub-themes | Codes | Verbatim examples |
| --- | --- | --- | --- |
| 1. Challenges and Barriers | **A. Financial, economic, and employment-related constraints** | Affordability issues; Financial Hardship; Accessibility of Care; Access Barriers; Cost of Care | *“I'm the epitome of most Canadians now I have a good job I make good money and I still can't afford anything...I would never ever even on a payment plan could I fathom having to put out that kind of money.” (P11)* |
|  | **B. Systemic challenges** | Resource Limitations; Accessing Healthcare; ODSP Barriers; Government Barriers; Inadequate Funding; Inequities in Access; Lack of Comprehensive Support; Insufficient Coverage; Navigating Government Assistance | *“Sometimes the healthcare system is really, really hard to navigate. Even for people in healthcare, it is absolutely so hard to navigate and get where you’re supposed to be and talk to the right person.”(P5)* |
|  | **C. Personal barriers to care** | Childhood Trauma; Impact of Early Experiences; Long-term Impact of Lack of Care; Lack of Dental Care in Childhood; Negative Past Experiences; Impact of Abuse; Lack of knowledge; Neglected Oral Health | *“I got depression really bad...my hygiene really declined...I didn't go anywhere didn't do anything...I was just depressed all the time and I didn’t want to see anybody.”(P13)* |
|  | **D. Stigma and discrimination** | Discrimination in Traditional Care Settings; Social Stigma; Judgmental Attitudes; Discrimination by Insurance: Drug Addiction; Marginalization | *“They (dental offices) don’t like seeing patients on government plans.” (P19)* |
| 2. Increased access to dental care through CSL program | **A. Appreciation for CSL** | Quality of Care; Program Satisfaction; Benefits of the Program; Positive Perception of CSL; Improved Patient Experience | *“It’s a very welcoming place...Everybody’s compassionate towards you. So it’s a good place to be...They took good care of me (…) I love this place.” (P12)* |
|  | **B. The CSL's expansion** | Desire for Expansion; Need for Program Expansion; Home-Based Care; Suggestions for Improvement: Critical Feedback | *“I’m wondering if something like that would even work in the future (...) that they have a list of people who need that they go to, you know each day, every week (…) just offering the basic care (at home).” (P5)* |
