# Supplementary material for: Addressing oral health equity through community service-learning and person-centered care in Ontario: patient and provider perspectives
Source: PLoS One. 2025 Oct 10;20(10):e0334089. doi: 10.1371/journal.pone.0334089 (PMC12513611; doi:10.1371/journal.pone.0334089)
Supplement: S3 Appendix — (DOCX) [file pone.0334089.s003.docx]

**Appendix 3**

**Table 4: Themes, sub-themes and codes, with verbatim examples from the HCPs (N=6).**

| Themes | Sub-themes | Codes | Verbatim examples |
| --- | --- | --- | --- |
| 1. Person-centred care | **A. Increased learner awareness of individualized circumstances** | Perspective-Taking; Understanding Patient Diversity; Recognizing Complexities | *‘‘Imagine waking up this morning in a tent. Do you really care that you have a dentist’s appointment in an hour? Or are you going to stay in that tent where it’s warm and dry? And the fact that there’s no phone for you to call to cancel or to reschedule I said that’s why there’s no-shows with the clients that I have. So explaining that to them when I was doing that the one day they were like that makes total sense.’’ (HCP 1)  ‘‘I think it broadens their horizon their awareness of kind of the challenges that folks encounter.’’ (HCP6)* |
|  | **B. Increased learner awareness of social determinants of health** | Eye-Opening Experience; Understanding Social Inequity; Awareness Of Disparity | *‘‘They know that these folks are out there to some extent they know they’re not getting served in the way they should’’ (HCP 6)  ‘‘I think it has opened their eyes to the severity of dental needs. And I’m hoping they do take this time to experience the challenges and barriers that people do have and that when they get into practice they have to understand that not everybody is the same.’’ (HCP 4)* |
