# Supplementary material for: Addressing oral health equity through community service-learning and person-centered care in Ontario: patient and provider perspectives
Source: PLoS One. 2025 Oct 10;20(10):e0334089. doi: 10.1371/journal.pone.0334089 (PMC12513611; doi:10.1371/journal.pone.0334089)
Supplement: S4 Appendix — (DOCX) [file pone.0334089.s004.docx]

**Appendix 4**

**Table 5: Shared themes, subthemes, and codes with verbatim examples from the patients and HCPs .**

| Themes | Sub-themes | Codes | Verbatim |
| --- | --- | --- | --- |
| 1. Supportive environment | **A. Increased empathy** | Understanding Client Barriers; Expanded worldview; Sense of Contribution | *“You got to realize what they’ve (patients) been through their lives and try to put yourself in their position.” (HCP6)* |
|  | **B. Emotional support** | Patient Trust; Building Trust; Addressing Anxiety; Emotional Barriers | *"They’re (patients) familiar with us they trust us and this is just one more service under the same roof. And they feel like they can trust that as well." (HCP2)* |
|  | **C. Dealing with anxiety and fear** | Dental Anxiety; Anxiety Management; Addressing Anxiety; Fear of Dentist; Concerns with Dental Procedures | *“I’d rather come back here and be comfortable then go to a new dentist, have my anxiety rise up to the top. There’s a difference, right?” (P6)* |
| 2. Patient empowerment and self-confidence | **A. For patients** | Empowerment; Improved Self-Esteem; Gratefulness for care; Impact on Patients; Positive Patient Transformation | *"And they had told me: 'I haven’t smiled in a long time' and now they feel comfortable to smile because they’ve been able to access that program." (HCP2)* |
|  | **B. Learners** | Developing Practical Solutions; Student Support Reception; Mandatory CSL; Clear Communication; Positive Change Through Education; Career Impact; Tangible Career Path; Reflective Practice; Powerful Reflections; Student Autonomy | *“I like that I’m helping them learn and help them get their degree right. And it’s gonna benefit them and it benefits me." (P15)* |
|  | **C. For community** | Comprehensive Support: Providing Holistic Care; Facilitating Access To Services; Quality Of Care; Holistic Approach; Improving Accessibility; Institutional Support | *“I really can't stress how important not only this clinic is to me but probably every single person that walks through that door...this community needs the dental clinic.” (P11)* |
